# From bingeing to cutting: the substitution of a mal-adaptive coping strategy after bariatric surgery

**DOI:** 10.1186/s40337-018-0213-3

**Published:** 2018-10-01

**Authors:** Louise Tækker, Bodil Just Christensen, Susanne Lunn

**Affiliations:** 1 Department of Psychology, University of Copenhagen, Copenhagen, Denmark; 2 Department of Food and Ressource Economics, University of Copenhagen, Copenhagen, Denmark

**Keywords:** Coping, Bariatric surgery, Binge eating, Self-harm, Eating disorder, Emotion regulation

## Abstract

**Background:**

An increase in self-harm emergencies after bariatric surgery have been documented, but understanding of the phenomenon is missing.

**Case presentation:**

The following case report describes a 26-year-old woman with obesity, who initiated self-harm behaviour after bariatric surgery. The patient reported that the self-harm was a substitute for binge eating, which was anatomically impeded after bariatric surgery.

Pre-surgical psychosocial assessment revealed Anorexia Nervosa in youth, which had later migrated to Binge Eating Disorder. At the time of surgery, the patient was not fulfilling the diagnostic criteria for Binge Eating Disorder because of a low frequency of binges. The remaining binges occurred when experiencing negative affect.

**Conclusions:**

Previous eating disorder pathology is an important consideration in pre-surgical assessments. For patients with affect-driven pre-surgical Binge Eating Disorder, therapeutic intervention before and after bariatric surgery could be indicated in order to secure the development of adaptive coping strategies. Furthermore, body weight as the only outcome measure for the success of surgery seems insufficient.

## Background

The following case report describes a 26-year-old woman with obesity, and a history of both Anorexia Nervosa (AN) and Binge Eating Disorder (BED), who was referred for bariatric surgery (BS). After surgery the patient successfully stopped binge eating but developed self-harming behavior as a means of regulating difficult emotions.

Disordered eating and non-suicidal self-harm behavior are well-known for clinical overlap, as an estimated 20% of individuals with eating disorders report comorbid self-harm [1]. Difficulties with regulating emotions have been suggested to serve as an underlying cause for both of these maladaptive behaviors [2]. Recent reports of an increase in self-harm emergencies after BS have been published [3, 4], but these offer little explanation as to why this increase in behavior can be observed after BS. Also, there seem to be no distinction between suicide attempts and non-suicidal self-harm behavior after BS. This case-report features one type of non-suicidal self-harm behavior, namely cutting, and will, from a psychological perspective, disclose one possible trajectory when initiating this type of self-harm behavior after bariatric surgery.

## Methods

The patient participated in a multidisciplinary research project, the so-called GO Bypass study [5]. The overall aim of the study was to identify multiple factors contributing to the variation in weight loss after BS, through a multidisciplinary approach. As such, the study followed patients for approximately two years, examined them in five study visits (at baseline, 1 week before surgery and 1.5, 6 and 18 months after surgery) and included psychological, physiological, socio-economic, genetic and other measures relevant for gaining knowledge about what constitutes a satisfactory BS process. For a more elaborate description of all of the segments of the GO Bypass study see Christensen & Schmidt 2018 [5].

In this case-report data from the following questionnaires have been included: Beck Depression Inventory (BDI-II) [6], Experiences in Close Relationship Scale (ECR) [7], the Eating Disorder Risk Composite (EDRC) of the Eating Disorder Inventory (EDI-3) [8], the Difficulty with Emotion Regulation Scale (DERS) [9] and the Hopkins Symptom Checklist revised (SCL90r) [10]. Also included from the GO bypass study are quotes from an anthropological interview as well as remarks and evaluations from the pre-surgical psychosocial assessment. All data has been gathered and analyzed independently from the clinical practice.

## Case presentation

At referral, the patient weighed 111 k (kg), and had a body mass index (BMI) of 41. A medical examination showed no organic illnesses or dysfunctions which could contraindicate surgery.

After the patient was accepted as a candidate for BS, she lost 8% of her excess weight by traditional dieting, in line with the local pre-surgical requirements, before a sleeve gastrectomy procedure was performed. At the time of surgery, the patient weighed 101 kg and had a BMI of 37. The pre-surgical weight loss, as well as the surgery and recovery period, had proceeded without medical or behavioural complications. At the 18 months follow-up, the weight-loss was 27 kg, corresponding to a new BMI of 31, and as a result the surgery and outcome could be deemed satisfactory.

The pre-surgical psychosocial assessment had revealed a severe history of both AN and BED. AN was of the restrictive type, with onset at age 16, minimum weight 45 kg and BMI 16.5. At age 18, the eating disorder migrated to BED, and the BMI rose accordingly, with the maximum weight being 110 kg and a BMI of 41. The patient was characterised by a high degree of body dissatisfaction, which had been present since childhood. As a consequence, the patient avoided scales as well as mirrors and public display. The patient reported she still had eating binges, but the frequency was low, with the most recent episode occurring three months prior to the psychosocial assessment interview. Within the past four years, she had not met the full diagnostic criteria for BED. Her existing eating binges were provoked when experiencing difficulty with emotion regulation, for example after a quarrel with her partner. Alongside the eating disorder symptoms, the patient suffered from depression. The symptoms were active at referral to BS but the patient was in treatment with antidepressants, which was described as helpful. Although the clinical impression was of depression in remission, the patient scored 36 on the Beck Depression Inventory (BDI-ii), corresponding to severe depression [6]. Family history revealed serious parental neglect, and childhood memories were considered “painful”, and as roots to the depressive inclination. Binge eating was described as something that could provide a feeling of security and comfort in a world of pain and chaos. Her current life situation was assessed as stable: the patient lived with her partner, with whom she planned to raise a family. The partner was characterised as a person who offered emotional support and had helped the patient gain healthier eating habits. The psychosocial assessment evaluated the patient as ‘at risk’ and in need for close monitoring, but did not recommend rejection of surgery. In addition, the pre-surgical psychosocial assessment concluded that since the patient was socially withdrawn and seemed very dependent on her partner, the continuation of the relationship was essential for a satisfactory surgery outcome. This vulnerable impression was supported by the following measures: the Experience in Close Relationships (ECR-r) questionnaire [7] showing an anxious-avoidant attachment pattern; the Difficulty with Emotion Regulation Scale (DERS) [9] which placed her at the 80th percentile (when using a median cut within the GO bypass study sample), and an alarming amount of psychiatric symptoms, measured with SCL-90-R [10]. See Table 1 for psychometric test-results.

**Table 1:** Pre- and post-operative development in psychometric scores

Measure	Baseline	18 month follow-up	Development
Psychiatric symptom load (GSI t score from SCL90r)	High (score 80)^a^	High (score 80)^a^	Unchanged
Depression (BDI-II)	Severe (score 36)	Moderate (score 24)	Improved
Difficulty in emotion regulation (DERS)	Score 96	Score 126	Aggravated^b^
Attachment style (ECR scale)	Anxious 4.0 Avoidant 1.6	Anxious 5.7 Avoidant 2.1	More anxious More avoidant
Eating disorder risk composite (t-score from EDI-3 subscale)	At risk (score 64)	Not at risk (score 39)	Improved
Bulimia Nervosa (t-score from EDI-3 BN subscale)	High (score 71)	Low (score 36)	Improved
Drive for thinness (t-score from EDI-3 DT subscale)	High (score 54)	Moderate (score 43)	Improved
Body dissatisfaction (t-score from EDI-3 BD subscale)	High (score 60)	Moderate-high (score 47)	Improved

GSI: Global severity index SCL90r: Hopkins symptom checklist 90 revised BDI-II: Beck Depression Inventory DERS: Difficulties in emotion regulation scale ECR: Experience in close relationships scale

EDI-3: Eating disorder Inventory-3 BN: Bulimia Nervosa DT: Drive for thinness BD: Body dissatisfaction

^a^Norms mean = 50, SD = 10

^b^No norms exist. Range is 36–180. Median cut within sample population is 69 at baseline, and 61 at 18 months follow-up

## Results

**Fig. 1:**
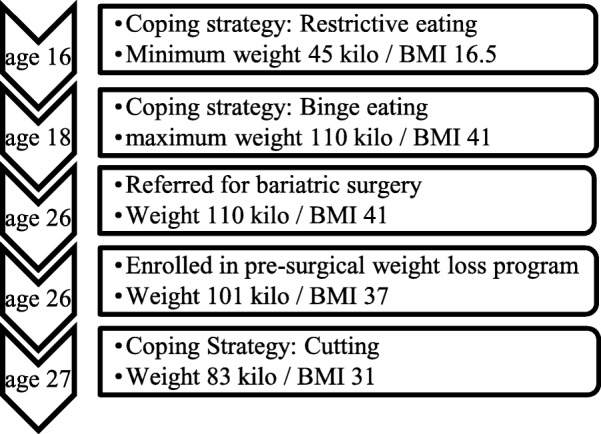
Timeline of weight and coping strategies

As part of the GO Bypass Study, five in-depth anthropological interviews were conducted in connection to each study visit. At the 18 months follow-up interview, the patient had been interviewed by the same anthropologist four consecutive times and had developed a trusting relationship with her. On this occasion, the patient spontaneously revealed that she had initiated cutting herself intentionally, with razor blades on both forearms several times a week. Approximately one year post-operative she had left her partner of seven years and the instability provoked by the break-up caused a relapse into depression. The following months were marked by anxiety, confusion and desolation, and culminated in two successive suicide attempts. The self-cutting began shortly after this and became aggravated with mental stress. The patient spoke out about how this behaviour functioned as a substitute for binge eating, which had been completely absent post-surgically. When asked about her former emotionally related eating behaviors, she explained the practice and context of the self-harm: *“It [self-cutting] was what I used very much instead of food. Earlier, I just ate you know […] it is this punishing myself because I don’t feel that I am worth anything, anyway. And at that time, I ate because this was what I could do back then […] It provides security, you know – that I have my razor blades. I use them and not the food - because I have gone off my food”*. For a timeline of the patient’s weight and coping strategies see Fig. 1.

Psychometric test results are listed in Table 1, which includes scores from the aforementioned questionnaires as well as from the Eating Disorder Inventory (EDI-3) [8]. The results show that while the patient’s overall psychiatric symptom load remains high, and well above the clinical case cut-off [10], symptoms of eating disorder and depression have improved notably after BS. However, the capacity to regulate emotions seems to have deteriorated and the attachment style is also more anxious and avoidant.

## Discussion

### Eating disorders and bariatric surgery

BED in BS candidates is quite common and its prevalence has been estimated to be between 10 to 27%, using DSM 5 criteria [11]. It is debated how pre-surgical BED relates to the outcome of the surgery since findings have pointed towards both poorer [12] and equally good long-term weight loss among pre-surgical BED patients compared to non-BED patients [13]. In line with these results BED is not considered as an absolute contraindication to BS. Instead, it is advised that the patient’s BED is assessed individually for severity and the possible consequences [14]. From psychotherapy research it has been shown that the subtype of BED in which binges are triggered by difficulties with regulating emotion indicates more severe symptoms as well as poorer therapy response than the subtype of BED in which binges are typically triggered by preceding restricted eating [15]. We hypothesise that the same differentiation is also at stake with regards to BS response, and that this might explain why outcomes of BS with pre-surgical BED have shown mixed results as the negative impact of subtypes vary.

### Mal-adaptive coping after bariatric surgery

In the research and clinical literature it has been argued that symptoms can substitute for each other, and that this can be an issue unless the underlying basic causes have been treated and cured [16]. When one compulsive behaviour seems to be replaced by another, it has been called ‘addiction transfer’ [17] ‘cross-addiction’ [18] and ‘symptom substitution’ [19]. With regards to BS, it has been debated whether or not patients are at risk of transferring their food addiction to other inexpedient behaviours post-surgery, as the procedure alters the anatomy and hence, to some extent, renders binge eating impossible. The focus in this area has mainly been on addictions related to diagnostic categories, such as alcoholism, drug-abuse or gambling [18, 20]. Behaviours on the margins of diagnoses, such as excessive shopping or sex addiction, have been briefly examined [20, 21], whereas non-suicidal self-harm behaviours, to our knowledge, have not been investigated.

It has been advocated that the three concepts; ‘addiction transfer’, ‘cross addiction’ and ‘symptom substitution’, should not be accepted as scientific phenomena since there is no evidence for a theoretical rationale of unresolved psychological problems causing one compulsive behaviour after the other [22]. In this case where the patient apparently substituted one symptom with another, we have therefore chosen to describe her behavior from a ‘coping’ perspective. Coping is a flexible process which has been defined as a conscious or subconscious cognitive and behavioral effort to master, tolerate or reduce conflicts [23]. Whereas ‘coping’ refers to the constructive solution of these conflicts or demands, ‘mal-adaptive coping’ describes strategies which might be effective on a short term basis, but offers no resolution of the conflicts or demands, either because the condition is unchangeable or beyond individual control. Mal-adaptive coping strategies can even be counterproductive to achieving permanent solution of the conflicts [24].

In the present case, we have insight into the thoughts and feelings of a patient both pre- and post-surgery due to the qualitative interviews conducted with her, and thereby into her reflections on the shift in her behaviour. Her statements show that the binge eating and self-harm serve the very same function, and this supports the notion of a shift in her maladaptive coping strategy, from bingeing to cutting, following BS. This substitution in symptoms may be accounted for by a common underlying mechanism behind BED and cutting, in which difficulty in emotion regulation plays a central role.

### Assessment, outcome measures and support

The case serves as a clear example of the insufficiency of measuring the weight loss and the absence of physical complications as the sole outcome criteria for success after BS. In the present case, no standard questionnaire or objective measure currently used in bariatric assessment would have uncovered the severity and nature of the patient’s symptoms. This underlines the importance of a thorough clinical pre-surgical psychosocial assessment, accompanied by a prolonged follow-up period as well as adequate therapeutic options for vulnerable patients.

## Conclusion

We presented a patient with a history of Anorexia Nervosa and Binge Eating Disorder who underwent bariatric surgery. The patient achieved satisfactory weight loss and had no obvious physical side effects or complications. A qualitative interview at the 18 months follow-up revealed that the patient had initiated cutting herself with razor blades as a substitute for binge eating. The case highlights a hitherto undescribed substitution of maladaptive coping strategies after bariatric surgery.

The transfer from binge eating to cutting can be summarised as follows: the patient’s eating disorder was driven by negative affect, but had in recent years been less active mainly due to a symbiotic relationship with a partner, in which the patient had been emotionally stabilised. When the relationship was terminated, the patient’s difficulty with emotion regulation resurfaced – this time with an obstruction to her ability to binge eat, which had previously been her primary emotion regulation strategy. Without the development of a more adaptive and expedient way of regulating emotions, the patient substituted one self-destructive way of handling emotions with another: from binge eating to cutting.

The case illustrates how weight as the only outcome measure following bariatric surgery is insufficient, and it stresses the need for psychosocial assessment and therapeutic interventions pre- as well as post-surgical. A case study such as this is suitable for generating hypothesis, but not for generalization.

More research is needed into the prevalence of non-suicidal self-harm after bariatric surgery, and also into how subtypes of Binge Eating Disorder, as well as the severity of past eating disorder pathology, relates to the outcome of bariatric surgery.
